# Anxiety-Related Coping Styles, Social Support, and Internet Use Disorder

**DOI:** 10.3389/fpsyt.2019.00640

**Published:** 2019-09-24

**Authors:** Sonja Jung, Cornelia Sindermann, Mei Li, Jennifer Wernicke, Ling Quan, Huei-Chen Ko, Christian Montag

**Affiliations:** ^1^Department of Molecular Psychology, Institute of Psychology and Education, Ulm University, Ulm, Germany; ^2^Student Counseling Center, Beijing University of Civil Engineering and Architecture, Beijing, China; ^3^Student Affairs Office, School of Life Science and Technology, University of Electronic Science and Technology of China, Chengdu, China; ^4^Department of Psychology, College of Medical and Health Sciences, Asia University, Taichung, Taiwan; ^5^Department of Medical Research, China Medical University Hospital, China Medical University, Taichung, Taiwan; ^6^The Clinical Hospital of Chengdu Brain Science Institute, MOE Key Laboratory for Neuroinformation, University of Electronic Science and Technology of China, Chengdu, China

**Keywords:** addiction, Internet use disorder, social support, social network, vigilance

## Abstract

**Objective:** The Internet can offer a seemingly safe haven for those being disappointed by relationships in the “offline world”. Although the Internet can provide lonely people with opportunities to seek for help and support online, complete withdrawal from the offline world comes with costs. It is discussed if people can even become “addicted” to the Internet. Of note, meanwhile, many researchers prefer the term *Internet use disorder* (IUD) instead of using the term “Internet addiction”. To illustrate the importance of one’s own social network supporting a person in everyday life, we investigated, for the first time to our knowledge, how social resources in terms of quality and quantity might represent a buffer against the development of IUD. Furthermore, anxiety related coping styles are investigated as a further independent variable likely impacting on the development of an IUD.

**Method:** In the present work, N = 567 participants (n = 164 males and n = 403 females; M_age_ = 23.236; SD_age_ = 8.334) filled in a personality questionnaire assessing individual differences in cognitive avoidant and vigilant anxiety processing, ergo, traits describing individual differences in everyday coping styles/modes. Moreover, all participants provided information on individual differences in tendencies toward IUD, the perceived quality of social support received, and the size of their social network (hence a quantity measure).

**Results:** Participants with larger social networks and higher scores in the received social support reported the lowest tendencies toward IUD in our data. A vigilant coping style was positively correlated with tendencies toward IUD, whereas no robust associations could be observed between a cognitive avoidant coping style and tendencies toward IUD. Hierarchical linear regression underlined an important predictive role of the interaction term of vigilance in ego-threat scenarios and perceived quality of social support.

**Conclusion:** The current study not only yields support for the hypothesis that the size of one’s own social network as well as the perceived quality of social support received in everyday life present putative resilience factors against developing IUD. It also supports the approach that special coping styles are needed to make use of the social support offered.

## Introduction

For some persons, the “offline world” is full of frustrations, mortifications, and disappointing relationships. This might be, in particular, the case for lonely, shy, and/or socially anxious individuals. For these groups of persons, the “online world” might offer a promising alternative with its abundant possibilities to cope with one’s own disappointments in everyday life [e.g., Refs. (1–6)]: In detail, social media and messenger applications offer possibilities to connect with other humans and to seek for approval and acceptance (3, 7, 8). Gaming platforms can provide fun but can also be a cathartic outlet to release aggression (9) and an escape from real-life challenges (10). From this perspective, for some individuals, the online world could be perceived as more attractive than the offline world. Therefore, the online world might resemble a safe refuge from disappointments of the offline life and may even function as a short-term remedy but with the risk of developing addictive Internet use [see, for example, *Model of Compensatory Internet Use* by Kardefelt-Winther (11)].

### On the Nomenclature Debate on “Internet Addiction”: Does the Term Internet Use Disorder Represent a Solution?

A potential diagnosis called “Internet addiction” has been discussed for more than 20 year [e.g., Refs. (12–14)]. Given the controversy with this term, please note that “Internet addiction” is mentioned in quotation marks in this work. And we explain in the following why we currently prefer the term *Internet use disorder* (IUD): In May 2019, the World Health Organization (15) ultimately ratified their decision to include *Gaming Disorder* as a distinct diagnosis in the *International Classification of Diseases, 11th Revision* (*ICD-11*) [see also Ref. (16)]. This diagnosis can be found under 6C51 in the category “disorder due to addictive behaviour” and can be diagnosed for online and offline gaming behavior. Of note, also, the American Psychiatric Association (17) included *Internet gaming disorder* in the *Diagnostic and Statistical Manual of Mental Disorders, Fifth Edition* (*DSM-5*) in 2013 (18). But in contrast to *ICD-11*, the term *Internet gaming disorder* was “only” included as an emerging disorder in its appendix but stimulated a lot of research. This was clearly of relevance for the recent decision to implement *Gaming Disorder* as an official diagnosis in *ICD-11*. Of importance, this new diagnosis might function as a blueprint for other online addictive behaviors, now. Using *Gaming Disorder* as a guideline, from our perspective, the research field has the chance to aim at a unification of nomenclatures used to study the field of online addictive behaviors. It is also noteworthy that the term *Internet use disorders* has been put forward in the Interaction of Person–Affect–Cognition–Execution (I-PACE) model by Brand et al. (19) [for a recent, update see Ref. (20)]. This term, on the one hand, orients itself toward the recent nomenclature *(Internet) Gaming Disorder* but also points toward the relevant point, that the pathological *use* of the Internet is at the center of psychologists’ and psychiatrists’ observations. The I-PACE model is a theoretical approach to describe the underlying processes of development and maintenance of IUD (19). The model describes the interplay between predisposing factors (e.g., neurobiological features and social cognition), moderators (e.g., coping styles), and mediators (e.g., affective and cognitive factors) as determinants of the development of specific forms of IUD (19). Perceived social support is sorted into the field of the predisposing factors that determine the core characteristics of a person; coping style is seen as a moderator in this process, underlining the important role of these two factors in the development of IUD (19).

Beyond the I-PACE model and recent developments in *DSM-5/ICD-11*, many researchers argue that it is better to use the term *problematic Internet use* (PIU) compared to “Internet addiction” in the literature [see a review by Yellowlees and Marks (21)]. Although this is valid from our perspective, the term PIU comes also with problems because it is not clear if PIU represents the end of the spectrum to be investigated or a transit zone for a person going from healthy *via* problematic toward pathological use. These arguments convince us to use, at the current moment, the term IUD. For a recent overview (also on neuroscientific aspects of IUD), see the review by Montag and Becker (22).

### Criteria and Prevalences of Internet Use Disorder

Possible diagnostic criteria for IUD were proposed, for example, by Tao et al. (23) (the term “Internet addiction disorder” was used in this work). Among others, they discussed symptoms like: preoccupation with the Internet, withdrawal when not being online for several days, tolerance, and difficulty controlling Internet usage behavior (23). The Gaming Disorder diagnosis, as a specific form of IUD, among others goes along with loss of control over gaming, continuing with gaming despite negative consequences, and, perhaps most important, significant impairments in private and/or business life due to excessive gaming (15). In the work of Müller et al. (24), 2.1% of a German sample (N = 2,512, aged 14–94 years) met criteria for IUD [but were diagnosed with the *Scale for the Assessment of Internet and Computer Game Addiction* (AICA-S) based on the *Skala zu Computerspielverhalten* (CSV-S), designed by Woelfling et al. (25)]. In the sample (N = 1,723 adolescent Germans, aged 14–17 years) of Wartberg et al. (26), even 3.2% showed signs of IUD [here measured with the *Compulsive Internet Use Scale* (CIUS) designed by Khazaal et al. (27)]. A higher prevalence of IUD for adolescents, for example, is in line with the work of Rumpf et al. (28) and Wu et al. (29), accentuating the importance of taking age into account as a relevant factor in IUD research. Aside from age, another important influence factor seems to be gender: males seem to be more vulnerable to developing IUD than females [see, for example, Refs. (29–31)]; but this view is more and more challenged given that one has to take a closer look at the specific forms of IUD.

### Unspecified and Specific Forms of Internet Use Disorder

As mentioned, beyond the broad term IUD, also, specific forms of IUD such as social media use disorder or Internet communication disorder (ICD) are currently hotly debated (32–37). In this realm, it is noteworthy that Montag et al. (38) already found support for the existence of different forms of IUD in a cross-cultural study. Testing the idea of Davis’ model on pathological Internet use (39), Montag et al. (38) observed that unspecified (then called *generalized*) IUD correlated to varying degrees with different forms of specific IUD. In the work by Montag et al. (38), the areas of excessive online pornography use, online video gaming, online shopping, and online social network use were covered. The unspecified form of IUD (including among others aimless browsing) was, in particular, highly correlated with ICD [see also a newer work by Müller et al. (40)]. This observed robust association between unspecified IUD and ICD may underline the importance of taking a closer look into social processes and social motivations (like the need for attachment, belonging, and connecting with others) to understand the developmental processes of IUD. In this context, it is also of relevance to see that mobile IUD (in the form of smartphone use disorder) is robustly associated with WhatsApp use disorder (41).

### On the Importance of Social Support in Human Life

Mikulincer and Shaver (42) postulated the idea of “*Homo auxiliator vel accipio auxilium* (one who helps or receives help)” (p. 8) (42). This idea focuses on the human need for (social) support, providing the band of *sapiens* with higher chances for survival, especially in childhood and adolescence (see also Bowlby’s attachment theory) (43, 44). Despite *sapiens*’ higher need for support in the early years of human life, the need for support and encouragement seems not to vanish in adulthood, but also, individual differences can be observed in its connection to different attachment styles [e.g., Refs. (45–47)]. In more modern psychoanalytic approaches, early attachment experiences are proposed to play a key role in the development of personality structure and psychopathology (as an example, see object relations theory [e.g., Ref. (48)]). In line with this view, addiction is described as an attachment disorder by some authors [e.g., Refs. (49, 50)], something also highlighted in affective neuroscience theory (ANT) by Jaak Panksepp (51). For an overview on selected principles of the Pankseppian ANT, see the recent work by Davis and Montag (52). Social bonds seem to play an important role in IUD, too. To illustrate this Milani (53) related Internet overuse in adolescents with dysfunctional coping strategies and dysfunctional interpersonal relationships. For links between Panksepp’s ANT and IUD, see the work by Montag et al. (54).

Social support and social network can be summarized as “social resource” [e.g., Ref. (55)] and are seen as potential protective or buffering factors in the context of psychological [e.g., Refs. (56, 57)] and/or physical health [e.g., Refs. (58, 59)]. Different effects of quantitative (social network) and qualitative facets (perceived social support) of social resource/interaction are also investigated individually in terms of being resilience factors for psychological distress (60). To illustrate this, in a meta-analysis Pinquart and Duberstein (59) described different effects of perceived social support and social network size (SNS) on cancer mortality when the variables of age and cancer type also have been considered. In the field of addiction, research on a potential protective role of the social resource concerning the onset of addictive behavior and a potential supportive role concerning withdrawal on one side and maintenance of abstinence on the other side has already been a matter of interest: For example, in a longitudinal study, Peirce et al. (61) described a buffering role of tangible social support with respect to the relationship between financial stress and the tendency to cope with alcohol. The positive outcome for maintenance of abstinence in members of Alcoholics Anonymous (AA) is often seen as a result of social support [see, for example, a review by Groh et al. (62)] and social network mechanisms (63). Nevertheless, we also mention that an “unhealthy” social network can also be a risk factor for addictive behavior: Schroeder et al. (64) found drug use in one’s social network to be a strong predictor for continuing drug use. These findings underline the importance of taking a closer look at network characteristics, too.

A study by Rotry et al. (65) also makes a case to distinguish between the quantitative and the qualitative facet of the social resource to understand psychiatric outcomes. Comparing patients still suffering from bulimia nervosa with individuals in remission from bulimia nervosa, they found that both investigated groups showed the same number of people in their network to offer advice, but individuals in remission had significantly more people in their network providing emotional support (65).

In the field of IUD, research on the protective role of the social resource is a matter of interest as well. Here, Zhang et al. (66) described a direct effect of subjective perceived support on the IUD dimensions *development of tolerance* and *time-management problems*. Furthermore, it is discussed in the literature if online forms of social support are comparable to offline forms of social support and if these online forms of social support may prove to be protective against the development of IUD. So far, results are contradicting, and mechanisms explaining such associations are not well understood [e.g., Refs. (67–70)].

### The Relevance of Taking Into Account Individual Differences in Coping Styles to Better Understand Internet Use Disorders

Alcohol, drugs, and unhealthy eating strategies are often used in a maladaptive way to cope with different forms of stress and challenges in life [e.g., Ref. (61)]. This might be, in particular, the case when everyday-used coping strategies or defense mechanisms prove to be insufficient, fail, or are maladaptive per se [e.g., Refs. (71–77)]. As a consequence, a closer look at individual differences in coping styles is of relevance to better understand protective and risk factors of IUD. Waqas et al. (78) described positive associations between the defense mechanisms projection, denial, autistic fantasy, passive aggression, as well as displacement and IUD scores, whereas they found negative associations between sublimation and IUD scores (78). Sublimation describes a mature form of defense where inacceptable instincts are redirected to a socially more approved behavior, for example, aggressive impulses are acted out in a creative way, for instance, by painting pictures [e.g., Ref. (79)]. In the current study, we investigated, for the first time to our knowledge, a potential role of the anxiety-related coping styles called vigilance and cognitive avoidance in the context of IUD [these coping styles are described in detail here: Refs. (80, 81)]. As a link between (social) anxiety and IUD often has been shown [see also a recent work (82)], a main focus on anxiety-related coping in the present work seems to be expedient [e.g., Refs. (1, 4, 83)]. The concept of these strategies is grounded in the psychoanalytic tradition with a close connection to the defense mechanisms proposed by Sigmund Freud (84) and his daughter Anna Freud (85). Vigilance describes a tendency to turn the attention to anxiety-provoking stimuli and an enhanced processing of them, whereas cognitive avoidance describes the tendency to turn the attention away from information that triggers anxiety (80). When connected with the psychoanalytic concepts of defense mechanisms, cognitive avoidance can be associated with repression or even suppression (considering the conscious character), whereas vigilance is associated with intellectualization (86, 87). We saw a closer link of cognitive avoidance with suppression than with repression. In the classic approaches to repression/sensitization that form the background of the cognitive avoidance/vigilance concepts we use in our work, an experiment with tachistoscopic presentation of emotional stimuli was used to measure unconscious processes (88). As we are working with self-report questionnaires, we cannot claim to measure unconscious processes; therefore, we believe that we did not measure repression but suppression. Suppression (in this context, cognitive avoidance) is seen as a mature defense mechanism that is used in a conscious way to suppress unwanted impulses (85, 79, 87). Intellectualization (in this context, vigilance) is seen as a more immature but neurotic form of defense that is usually seen as a defense mechanism prominent in adolescence (85, 87). The idea that defense mechanisms change with age [e.g., Ref. (89)] falls into the debate asking if different coping styles prevail in different age groups [e.g., Refs. (90–93)].

Vigilance (or *sensitization*) (94) is often found to be associated with undesirable psychological outcomes such as higher anxiety scores [e.g., Refs. (95, 96)], higher self-reported stress [e.g., Refs. (97, 98)], or higher degrees of depression [e.g., Ref. (99)]. In contrast, a cognitive avoidant copying style (or *repression*) (94) has been associated with lower anxiety, stress, and depression scores [e.g., Refs. (95, 97, 99)]. Gender differences in the use of coping strategies have been described earlier [see a meta-analytic review by Ref. (100)]. Among others, Egloff and Krohne (80) and Jung et al. (101) observed significantly higher vigilance scores for women compared to males, whereas male participants showed significantly higher cognitive avoidance scores than female participants.

As vigilance seems to be associated with a higher psychological vulnerability (stress, depression, anxiety; see above), we expect, in our first hypothesis, a vigilant coping style to be associated with higher tendencies toward IUD, whereas a cognitive avoidant style (comparable to the mature defense mechanism of suppression) should be associated with lower tendencies toward IUD. This would be in line with the results of Waqas et al. (78), who only found a negative association with IUD for the mature defense mechanism of sublimation.

As a second hypothesis, we expect high perceived social support and a larger social network to be associated with lower tendencies toward IUD.

In addition, and as a third hypothesis, we propose that the associations between coping styles and IUD are influenced by the different forms of social resource. In detail: We propose an interaction effect of anxiety-related coping styles and the social resource on IUD. This means that a positive association of higher vigilance and higher IUD can be softened by high perceived social support and a larger social network. In contrast, the proposed negative association between a higher cognitive avoidant coping style and lower IUD is expected to be even stronger for individuals with high social support and larger SNS. In sum, we expect that individuals scoring higher on cognitive avoidance together with available social resources show the lowest tendency to develop an IUD. In line with the findings of Rotry et al. (65), we expect a higher impact of social support (quality) than of SNS (quantity) on IUD.

## Methods and Materials

### Participants

#### German Sample

N = 581 participants (n = 165 males and n = 416 females; Mage = 23.165; SDage = 8.253), mostly students (85.71%), gave electronic informed consent and completed the following self-report questionnaires: *“Angstbewältigungs-Inventar*” (ABI, English: Anxiety Coping Inventory) (80), Generalized Problematic Internet Use Scale 2 (GPIUS2) (102), the social support subscale of the “*Fragebogen zur Erfassung von Ressourcen und Selbstmanagementfähigkeiten*” (FERUS, English: Questionnaire Assessing Resources and Self-Management Skills) (103), and a single-shot item measuring the size of the social network (104). Four participants were younger than 18 years. Those participants provided a declaration of consent of their legal guardian additional to the standard informed consent. All participants are part of the Ulm Gene Brain Behavior Project (UGBBP). The study was approved by the local ethics committee at Ulm University, Ulm, Germany. Fourteen participants had to be excluded due to missing questionnaire data. The final data set consisted of 567 participants (n = 164 males and n = 403 females; M_age_ = 23.236; SD_age_ = 8.334).

#### Chinese/Taiwanese Samples

It was also planned to recruit a sufficiently large Asian sample. Unfortunately, this did not work. N = 104 from Taichung, Taiwan, and N = 34 from Beijing, China, could be recruited. Unfortunately, these samples are too small in terms of power analysis to detect the expected effects (we also do not want to mix the participants from both sites given differences in the characters applied in the questionnaire etc.).

For complete transparency, we provide readers with descriptive statistics and Spearman correlations between GPIUS2, vigilance, cognitive avoidance, social support, social network, and age in the Taiwanese sample, which are provided in the **Supplementary Material**. This Taiwanese sample is, at least, beyond 100 participants. The reader will see that findings are, in part, in line with what we observe in the German sample. Some differences might occur due to the smaller power for statistical testing. Finally, some findings might not be replicated given the differences in the cultural background of Germany and East Asian culture. That said, with the appropriate power available, we often were able to replicate findings across Western and Eastern sites [e.g., Refs. (105, 106); even when taking into account biological variables, see Ref. (107) or (108)]. For an overview on tackling the replication crisis in psychology using cross-cultural work, see a recent work by Montag (109).

We want to mention that the questionnaires for data collection in China and Taiwan have been back-and-forth translated by bilingual Chinese-speaking psychologists and yielded acceptable internal consistencies (Cronbach’s alpha ranging between .660 and .927). If translations of these questionnaires are needed, we are happy to share them with the community.

### Questionnaires

#### Generalized Problematic Internet Use Scale 2 (GPIUS2)

IUD was assessed *via* the 15-item-long GPIUS2 (102). This instrument consists of the following five subscales: *Preference for Online Social Interaction*, *Mood Regulation*, *Cognitive Preoccupation*, *Compulsive Internet Use*, and *Negative Outcome*. Each subscale consists of three statements, e.g., “*I prefer communicating with people online rather than face-to-face”*. All items are rated on an eight-point Likert scale indicating the degree of agreement from 1 = “definitely disagree” to 8 = “definitely agree”. For further analyses an overall index was calculated *via* a sum score. A good internal consistency with a Cronbach’s alpha of .900 was found in the German sample. Note that this version of the questionnaire has been used also in older works from our group, such as Montag et al. (38) and Peterka-Bonetta et al. (110).

#### Angstbewältigungs-Inventar (ABI)

To measure individual differences in the coping styles of vigilance and cognitive avoidance, we used a stimulus–response inventory in both samples. German participants filled in the ABI (English: anxiety coping inventory) (80), the German version of the *Mainz Coping Inventory* (MCI) (111). The inventory consists of two subscales: ABI-E (four ego-threat scenarios, e.g., a job interview) and ABI-P (four physical threat scenarios, e.g., visiting the dentist). For each of the different scenarios [all used scenarios can be found in Ref. (111), in English language], participants need to rate which of 10 different coping styles they would use (1 = “applicable” or 0 = “not applicable”). For each fictitious scenario, five vigilant (e.g., “information search” or “anticipation of negative events” (p.192) (80)) and five cognitive avoidant coping strategies (e.g., “diversion” or “trivialization” (p. 192) (80)) are presented to allow separate assessments of the coping styles: vigilance in the ego-threat scenario (VIG-E) as well as the physical threat scenarios (VIG-P) and cognitive avoidance in the ego-threat scenarios (CAV-E) and in the physical threat scenarios (CAV-P). Total scores for both styles can be calculated: a total score for vigilance (VIG-T) and a total score for cognitive avoidance (CAV-T). The internal consistencies of all subscales and the total scores were acceptable. Cronbach’s alphas lied between .734 and .855 in the German sample.

#### Fragebogen zur Erfassung von Ressourcen und Selbstmanagementfähigkeiten (FERUS)/Social Support Scale

In order to assess the construct of social support, we used a slightly adjusted version of the FERUS (English: questionnaire assessing resources and self-management skills) Social Support Scale (103). The scale was once adjusted for a study conducted with cancer patients in our department; that version was used in this study, too. Only one FERUS item was slightly changed in our adjusted version (“(…) if I am ill” was changed to “(…) if I don´t feel well” with respect to the situation of cancer patients). As the changes were only minor and we already checked reliability of the German and Chinese versions of this adjusted FERUS, we found it reasonable to use this version in our current study, as well. The FERUS (103) is a German questionnaire to assess individual *resources*, like *social support* and *motivation to change*, as well as skills in *self-management*, like *coping, introspection, self-efficacy, self-verbalization*, and *hope*. This self-report is generally used to detect psychotherapeutic progress. For the current study, only the Social Support Scale of the FERUS was used. This scale consists of 10 items designed as statements concerning the helpfulness of the social background of the individual, e.g., *“If I want to talk about a problem, I know to whom I can go”*. The degree of consent to each statement is rated on a five-point Likert scale from 1 = "not true" to 5 = "very true". A sum score of all 10 items was calculated. An excellent internal consistency with a Cronbach’s alpha of .910 was found in the German sample.

#### Social Network Size

To assess the size of one’s social network, we used the following single-shot item: *“How many people do you have near you that you can readily count on for help in times of difficulty, such as watch over children or pets, give rides to hospital or store, or help when you are sick?”*. The item is based on the work by Blake and McKay (104) and was used, for example, by Koopman et al. (112) as a single item measure of social support.

In the current work, it is used as a measure for the size of the social network as its focus lies more on the quantitative than the qualitative aspect of the social resource. Participants had to state whether they have “0”, “1”, “2 to 5”, “6 to 9”, or “10 or more” close people they can count on.

### Statistical Analyses

Both the inspection of the histograms and the Shapiro–Wilk tests (*p* < .001) indicated non-normal distributions of the GPIUS2 overall score. Therefore, a Blom-rank transformation was carried out. The Blom-transformed GPIUS2 overall score was used in all conducted analyses (for histograms of the distribution of the GPIUS2 scores before and after Blom transformation, see **Supplementary Material**). For correlations between the GPIUS2 subscales, social support, social network, coping styles, and age, please see **Supplementary Material**. Hierarchical linear regression was carried out for all variables that were associated with the GPIUS2 scores and all interaction terms that conformed to the hypotheses. For reasons of conciseness and comparability, two regression models are calculated, one regarding social support (Model 1) and one SNS (Model 2). The regression analyses were conducted block-wise. The first block consisted of age and gender, the second block consisted of all coping variables and social resource variables (in Model 1: FERUS score for social support; in Model 2: dummy-coded SNS) that were correlated with the GPIUS2 score, and the third block consisted of interaction terms with relevance to our hypotheses. The SNS variable was included dummy-coded in the regression model, with “0 people” as the reference group. To take the problem of multiple testing into account, confidence intervals (CIs) were calculated using bootstrap analysis, which were bias corrected and accelerared; 1000 samples). When significant interaction terms could be observed, we used median splits of the respective data to allow an easy interpretable graphical presentation of the main results.

As general alpha level of .05 was used. All statistical analyses were conducted using SPSS 24.

#### Preselection of the Potential Predictors

A preselection of potential predictors to include in the regression models was conducted *via* correlation analyses (Spearman correlation) and, in the case of gender, with a t-test.

The potential predictors were only included into the regression models if significant associations with the transformed GPIUS2 score were found. All tests were performed two-tailed.

## Results

### Descriptive Statistics

Mean scores and standard deviations of the GPIUS2, vigilance, cognitive avoidance, social support, and SNS are presented in **Table 1**.

**Table 1:** Mean scores (standard deviations) of GPIUS2, all ABI variables, social support and SNS, and percentages for each SNS category for the total sample and for the male and female subsamples with mean differences and Cohen’s *d*.

	Total (N = 567)	Male (n = 164)	Female (n = 403)	*MD*	*Cohen’s d*
GPIUS2	37.07 (16.20)	39.76 (17.46)	35.98 (15.55)	3.78	.23
CAV-E	10.09 (4.04)	11.37 (3.95)	9.56 (3.96)	1.81	.46
CAV-P	12.11 (3.63)	12.93 (3.40)	11.77 (3.67)	1.16	.32
CAV-T	22.19 (6.58)	24.30 (6.43)	21.34 (6.45)	2.97	.46
VIG-E	13.73 (3.90)	12.62 (4.15)	14.18 (3.71)	–1.57	.41
VIG-P	10.47 (4.02)	8.99 (3.87)	11.07 (3.93)	–2.08	.53
VIG-T	24.20 (6.95)	21.61 (7.17)	25.26 (6.58)	–3.65	.54
Social support	44.84 (5.97)	43.01 (7.13)	45.59 (5.25)	–2.59	.44
SNS (score)	3.32 (0.72)	3.27 (0.71)	3.34 (0.72)	–.07	.10
SNS (0)	0.88%	1.83%	0.50%	–	–
SNS (1)	4.76%	3.66%	5.21%	–	–
SNS (2–5)	62.61%	65.85%	61.29%	–	–
SNS (6–9)	24.51%	22.56%	25.31%	–	–
SNS (10 or more)	7.23%	6.10%	7.69%	–	–

GPIUS2, Generalized Problematic Internet Use Scale 2; ABI, Angstbewältigungs-Inventar; CAV-E, cognitive avoidance (ego threat); CAV-P, cognitive avoidance (physical threat); CAV-T, cognitive avoidance (total score); VIG-E, vigilance (ego threat); VIG-P, vigilance (physical threat); VIG-T, vigilance (total score); SNS, social network size; MD, mean difference.

GPIUS2 scores are untransformed in this table for easier interpretation. Percentages do not add up to 100% in the total sample due to rounding inaccuracies.

### Preselection of Predictors

A significant effect of gender on the GPIUS2 score was found (*t*(565) = 2.382, *p* = .018) with males having higher scores than females. Significant positive correlations with the GPIUS2 score were also found for all vigilance variables (VIG-E, VIG-P, VIG-T), and significant negative correlations with cognitive avoidance in the ego-threat scenario (CAV-E), social support (FERUS), SNS, and age. Spearman correlations are presented in **Table 2**. Correlation coefficients of the putative prognostic variables that are taken into the model are bolded.

**Table 2:** Spearman correlations between GPIUS2 (Blom-transformed), cognitive avoidance (CAV), vigilance (VIG), social support, social network size (SNS), and age in the German sample (N = 567).

Correlation coefficients										
	GPIUS2	CAV-E	CAV-P	CAV-T	VIG-E	VIG-P	VIG-T	Social support	SNS	Age
GPIUS2	1	−.105*	−.026	−.086*	.160***	.127**	.164***	−.340***	−.137**	−.136**
CAV-E			.437***	.866***	−.373***	−.172***	−.318***	.017	.024	.054
CAV-P				.811***	−.094*	−.388***	−.277***	.049	.073	−.076
CAV-T					−.286***	−.317***	−.350***	.043	.059	−.013
VIG-E						.509***	.863***	−.043	−.068	−.017
VIG-P							.865***	−.039	−.088*	−.018
VIG-T								−.044	−.088*	−.016
Social support									.345***	−.039
SNS										−.057

*p < .05; **p < .01; ***p < .001 (two-tailed tested). Potential predictors are highlighted by bolded correlation coefficients. Only subscales of the ABI were considered as potential predictors.

The first variables entered into the regression model as potential predictors were age and gender. In the second block, vigilance (VIG-E, VIG-P), cognitive avoidance (CAV-E), and social support (Model 1) or dummy-coded SNS (Model 2) were included. In the last block, the interaction terms of cognitive avoidance (CAV-E) with social support (Model 1) or dummy-coded SNS (Model 2) as well as the interaction terms of vigilance (VIG-E and VIG-P) with social support (Model 1) or dummy-coded SNS (Model 2) were included in the respective model.

### Hierarchical Linear Regression

#### Model 1

Hierarchical linear regression showed for the Model 1 [Block 1 + Block 2 + Block 3] including all potential predictors the highest adjusted *R* *^2^* = .266 (*F*(9, 557) = 23.747, *p* < .001). The first block (Model 1 [Block 1]) including only age and gender (adjusted *R* *^2^* = .114) and the Model 1 [Block 1 + Block 2] including CAV-E, VIG-E, VIG-P, and social support (adjusted *R* *^2^* = .258) showed smaller adjusted *R* *^2^*s compared to the Model with all three blocks and therefore a minor prediction of the GPIUS2 score. The changes in *R* *^2^* between Model 1 [Block 1] and Model 1 [Block 1 + Block 2] (*p* < .001) and Model 1 [Block 1 + Block 2] and Model 1 [Block 1 + Block 2 + Block 3] (*p* = .029) were significant. The results for each potential predictor in the Model 1 [Block 1 + Block 2 + Block 3] are listed in **Table 3**. Significant predictors are bolded. Bootstrapping analysis verified the significance of age (CI: [−.045; −.033]), gender (CI: [−.409; −.107]), VIG-P (CI: [.006; .202]), social support (CI: [−.419; −.261]), the interaction term of VIG-E and social support (CI: [.017; .247]) with the GPIUS2 variable.

**Table 3:** Model 1 [Block 1 + Block 2 + Block 3]: hierarchical linear regression with GPIUS2 score as dependent variable and age, gender, cognitive avoidance, vigilance, social support and the respective interaction terms as potential predictors.

Variable	*B*	*SE of B*	*Beta*	*P*	*CI*
**Age** **^a^**	−.326	.036	−.331	<.001	[−.045; −.033]
**Gender** **^a^**	−.254	.084	−.117	.003	[−.409; −.107]
CAV-E	−.062	.039	−.063	.115	[−.144; .015]
**VIG-E**	.090	.045	.091	.045	[-.005; .173]
**VIG-P** **^a^**	.103	.043	.104	.018	[.006; .202]
**Social Support** **^a^**	−.337	.037	−.342	<.001	[−.419; −.261]
CAV-E × Social Support	.048	.032	.059	.132	[−.032; .114]
**VIG-E × Social Support** **^a^**	.120	.048	.121	.012	[.017; .247]
VIG-P × Social Support	−.024	.042	−.027	.568	[−.118; .048]

All predictors except gender in z-standardized form; gender coded: 1 = male, 2 = female; ^a^significant after bootstrapping analysis, such significant predictors are presented in bold letters.

**Figure 1:**
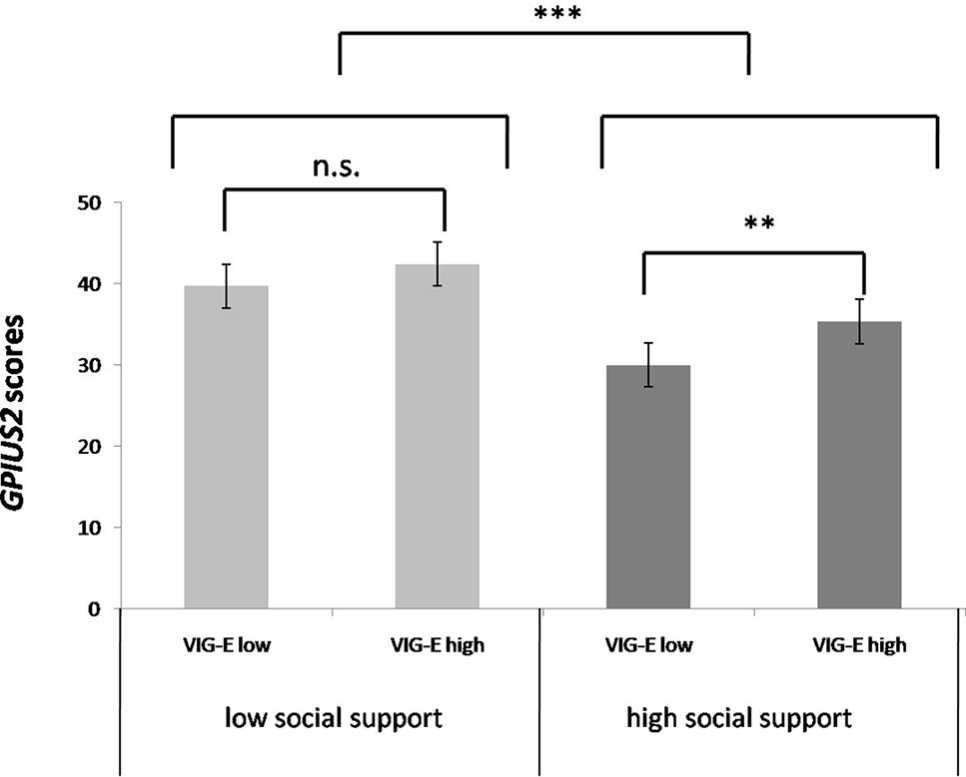
GPIUS2 scores according to social support and vigilance in the ego-threat scenario [brackets indicating significance of group differences; **p < .01 for median-splited vigilance in the group of high social support; ***p < .001 for the main effect of social support; derived from *post-hoc* test; n.s., not significant; error bars indicate -/+ 1 standard error].

To facilitate interpretation of the interaction between VIG-E and social support, a graphic (**Figure 1**) was designed using median splits for the variables social support and VIG-E.

#### Model 2

Hierarchical linear regression showed for Model 2 [Block 1 + Block 2] including age, gender, CAV-E, VIG-E, VIG-P, and dummy-coded SNS the best prediction for the GPIUS2 score, with an adjusted *R* *^2^* = .173 (*F*(9,557) = 14.183, *p* < .001). The adjusted *R* *^2^* of Model 2 [Block 1] with age and gender as predictors was .114, and the adjusted *R* *^2^* of the Model 2 [Block 1 + Block 2 + Block 3] with all potential predictors was .171. The changes in *R* *^2^* between Model 2 [Block 1] and Model 2 [Block 1 + Block 2] (*p* < .001) were significant, whereas the changes in *R* *^2^* between Model 2 [Block 1 + Block 2] and Model 2 [Block 1 + Block 2 + Block 3] (*p* = .583) were not significant. The results for the potential predictors of the Model 2 [Block 1 + Block 2] are listed in **Table 4**. Significant predictors are bolded. Bootstrapping analysis verified the significance of age (CI: [−.046; −.032]), gender (CI: [−.558; −.225]), VIG-P (CI: [.014; .194]), SNS (2–5 individuals) (CI: [−1.351; −.271]), SNS (6–9 individuals) (CI: [−1.590; −.444]), and SNS (10 or more individuals) (CI: [−1.526; −.398]).

**Table 4:** Model 2 [Block 1 + Block 2]: hierarchical linear regression with GPIUS2 score as dependent variable and age, gender, cognitive avoidance, vigilance, and dummy-coded SNS as potential predictors.

Variable	*B*	*SE of B*	*Beta*	*P*	*CI*
**Age** **^a^**	−.332	.038	−.337	<.001	[−.046; −.032]
**Gender** **^a^**	−.387	.087	−.178	<.001	[−.558; −.225]
CAV-E	−.073	.041	−.074	.075	[−.159; .015]
**VIG-E**	.096	.048	.097	.045	[−.011; .195]
**VIG-P** **^a^**	.109	.046	.111	.017	[.014; .194]
SNS (1)	−.525	.439	−.114	.231	[−1.217; .213]
**SNS (2–5)** **^a^**	−.833	.406	−.409	.041	[−1.351; −.271]
**SNS (6–9)** **^a^**	−1.039	.410	−.454	.012	[−1.590; −.444]
**SNS (10 or more)** **^a^**	−.970	.426	−.255	.023	[−1.526; −.398]

All predictors except gender in z-standardized form; gender coded: 1 = male, 2 = female; ^a^significant after bootstrapping analysis; number of people in the social network in parentheses. Significant predictors are presented in bold letters.

## Discussion

The aim of the current study was to investigate the role of anxiety-related coping styles in the development of IUD taking into account the qualitative and quantitative facets of the social resource. We did not find the proposed negative associations between cognitive avoidance and IUD in a hierarchical linear regression. Nevertheless, these results are in line with Waqas et al. (78), who only observed a relevant negative association between IUD and sublimation but no other mature defense style measured *via* the Defense Style Questionnaire (DSQ-40) (113). Especially, no associations between suppression (comparable to the here-measured cognitive avoidance) and IUD were found in their work. Instead, we detected significant positive correlations between IUD and vigilance in both scenarios. This is in line with our hypothesis. Individuals with tendencies to mainly cope in a vigilant way seem to be more vulnerable, e.g., to stress, depression, and anxious reactions [e.g., Refs. (95, 97, 99)]. Associations between IUD and, e.g., social anxiety [e.g., Refs. (1, 83)], stress [e.g., Ref. (114)], and depression (115) have been described before. It is imaginable that anxiety-related coping styles play a key role in IUD comorbidity and are also of importance in the understanding of risk factors and the development of therapeutic approaches. Moreover, individuals easily feeling unease in situations that could cause physical harm or challenge one’s own self-worth might show a tendency to flee into physically harmless adventures of the online world. Interaction with real people and real-world social situations could be seen as potential physical or self-worth-challenging dangers. Potential harmful social interactions are therefore something that should be avoided and not something that can be experienced as helpful or supportive. In line with this idea, Lee and Stapinski (4) described the importance of *fear of negative evaluations* during face-to-face contacts for a better understanding of the positive association between social anxiety and IUD. Moreover, in the model proposed by Caplan (116), individuals with deficits in social skills are described to prefer online interactions above face-to-face contact.

As proposed, we found negative correlations between social support and IUD, as well as a negative association between SNS and IUD. This may underline the potential protective character of the social resource in the development of IUD. As expected, the detected correlation for social support and IUD was stronger than the correlation found for SNS and IUD (social support: −.340 vs. SNS: −.137), supporting the idea of a special role of perceived social support in comparison to SNS in the search for protective factors. This result is in line with the work of Rorty et al. (65). In the regression models, the model with social support (Model 1) as well as the model with SNS as a predictor (Model 2) showed significant results. We were not able to find any interactions between vigilance measured in the physical threat scenario and the variables dealing with social resources. But availability of social resources (in terms of quality) seems to play a different role for individuals with a tendency to cope mainly vigilant in situations where the self/ego is challenged. We only found an interaction for vigilance in the ego-threat scenario and social support (see Model 1). These results may again underline the proposed superordinate role of social support when compared to SNS. Taking a closer look at this interesting interaction term, it seems as if individuals with the lowest tendencies to cope in a vigilant way in ego-threat scenarios can benefit the most from the social support that is offered to them. Maybe a focus on the anxiety-provoking content of a situation makes the individual kind of blind to available (social) help in this situation (80). To understand this interaction, a closer look at the maturity of the defense mechanism seems to be a necessary step. Defense mechanisms are described to change across the life span [e.g., Ref. (89)] and are especially associated with mental disorders, when used rigidly (e.g., 117) and not in accordance with the current developmental step (79). Malone et al. (118) already underlined the importance of defense maturity in the building of social bonds. Therefore, whether offered social support can be used in a helpful way by an afflicted person seems to depend on the kind of defense mechanism/coping strategy used. Malone et al. (118) observed a partially mediating role of social support in the association between adaptive (mature) defenses and physical health. In contrast, our data are more supporting the idea of a moderating role of the coping strategies proposed in the I-PACE model (18). Mature defense mechanisms are needed to ask for, accept, and profit from social support. That is where psychotherapy comes into play. The change of defense mechanisms can be seen as an aim or one of the positive effects of psychodynamic psychotherapy [see, for example, Ref. (119)]. Our results may underline the importance of psychodynamic psychotherapeutic approaches not only in the treatment of IUD but also in the prevention of it. A closer look at different coping styles could represent a promising new approach in the development of screening tools and therapeutic manuals because current existing therapeutic approaches and manuals for the treatment of IUD are mostly grounded in cognitive behavioral therapy (CBT) [e.g., Refs. (120–125)]. In line with this, so far, only a few empirical works have dealt with psychodynamic approaches in the treatment of IUD [see, for example, a case study by Essig (126), and a multi-centric study by Lindenberg et al. (127)]. In sum, diagnosing dominant coping styles/defense mechanisms (e.g., *via* specialized screening tools) could help to detect persons at high risk to develop an IUD.

Another promising therapeutic approach to treat persons afflicted with IUD could be the inclusion of mindfulness-based concepts where patients learn, among other things, to control and focus their attention in a conscious way [e.g., Ref. (128)]. Individuals with tendencies to cope mainly vigilant may be able to learn through mindfulness to willingly remove their focus from anxiety-provoking material to the available help and support from one’s own peer group. Of note, observations of Arslan (129) are in line with this idea, demonstrating the importance of mindfulness as a mediator between psychological maltreatment (here: experience of psychologically abusive parental behavior) and IUD (129). Beyond that, mindfulness-based interventions have already been described to be a promising therapeutic approach in the broader field of behavioral addictions [e.g., Ref. (130)].

The maturity of defense mechanisms can additionally offer an explanation of why adolescents seem to be more vulnerable to developing IUD (26, 131), as Anna Freud (85) saw intellectualization (in our work, comparable with vigilance) as a defense mechanism of adolescence. Using social support personally in a helpful way might be something to be learned first.

A limitation of the current study is the unbalanced sample composition with respect to age, gender, and professional background. As we expect defense mechanisms to change with age [e.g., Ref. (132)], a sample with a broader age range, with the possibility to build different age categories, would have helped to confirm the idea of an association between defense maturity and IUD. In addition, a balanced sample with respect to gender and professional background would confirm generalizability of the results. Furthermore, gender differences in the association between social support and IUD have been described by Yeh et al. (133) to be mediated by depressive symptoms. A gender-balanced sample should therefore include psychological symptoms to consider these clinically important potential mediation.

Furthermore, we did not take a closer look at social network characteristics, even though we did mention possible negative consequences of an unhealthy network in the context of addictive behaviors [e.g., Ref. (64)]. Future studies concerning the association between social network and IUD should take a closer look at these characteristics. It seems to be possible that an unhealthy network (e.g., consisting of somehow less supportive individuals), perhaps also with addictive tendendiens toward IUD vs. a healthy network (e.g., consisting of individuals with low addictive tendencies and caring characters) can end up in completely contrary effects. In addition, we mention that the item of Blake and McKay (104) measuring SNS in our work strongly focuses on assessing people in one’s own social network with close physical proximity to the individual. Therefore, it is possible that applying a broader concept of the term *social network* in the administered item of interest would have led to different results (e.g., “If I am sad, I have someone I can call”).

Another limitation of our study is our focus on offline social support. Online social support has been described, for example, by Leung (68) to be comparable to offline social support in its stress-buffering capacity in adolescents and children. In line with that, Ybarra et al. (70) pointed out the importance of both online and offline social support in the group of lesbians, gays, bisexuals, and transgenders. They found different positive effects of both online and offline social support, discussing that one cannot be simply replaced by the other (70). In contrast, Hardie and Tee (67) reported a higher profit from social support *via* Internet social networks for Internet over-users. That said, we need to mention that the FERUS items used to assess social support in the present work (and the item assessing the SNS) is formulated in a broad sense. Hence, some participants might have thought about their lives in the online and/or offline world, while answering the items. Again, future studies need to implement more inventories explicitly asking about social support/SNS in the online and offline world. Without such a clear distinction, researchers will not be able to carve out potential different effects of online vs. offline social support. Beyond that, associations between (online and offline) social support and IUD ultimately will be influenced by many mediating and moderating factors. The aforementioned I-PACE model by Brand et al. (19, 20) gives a good overview on the complex nature of IUD and how moderators and mediators might impact on the development of IUD. As mentioned earlier in this work, according to Brand’s model, such moderators/mediators involve the here-investigated coping styles but also the range of affective and cognitive factors.

Moreover, we have to point out the correlative nature of our work. Therefore, the reported associations prove no causality, and longitudinal studies are needed to follow up on the here-presented results. Even though the interaction between vigilance (ego-threat) and social support is very interesting and fits into the psychodynamic approach, we must highlight that the intake of the interaction terms in Model 1 only improved the explained variance (as compared to Model 1 [Block 1 + Block 2]) for 1%. Furthermore, all variables that were taken into account were measured *via* self-report questionnaires. This approach comes with its own limitations; especially in the case of the measurement of anxiety-related styles, we must assume moderate reflective functioning of the participants and consciousness of the strategies. In the case of the measure concerning the assessment of IUD, we again assume a certain ability level on the participant’s side to reflect on one’s own life. In particular, in the early phase of developing addictive tendencies toward the Internet, it is questionable that such abilities can be expected. Finally, the readers will see that the data set from East Asia did not support all of our present conclusions based on the German sample. That said, the many limitations coming with this data set are presented both in the methods and in the **Supplementary Material**. Still, we believe it to be of importance for reasons of transparency to report these data.

Despite these limitations, we believe that our study is able to underline the importance of quality of social support and special forms of coping in the search for protective factors to not suffer from IUD. As demonstrated, it might not be sufficient to just have social resources at hand. A person’s individual needs, the right strategies to actually profit from the support offered, and quality seem to be more important than mere quantity.

## Data Availability

The datasets generated for this study will not be made publicly available; not all participants gave consent for sharing the data.

## Ethics Statement

The studies involving human participants were reviewed and approved by Ethics committee at Ulm University, Ulm, Germany. Written informed consent to participate in this study was provided by the participants’ legal guardian/next of kin.

## Author Contributions

SJ and CM designed the present study. SJ drafted the first version of the manuscript, which was revised by CM. SJ carried out all statistical analyses, which independently were checked by CS and JW. The German sample was collected by SJ, CS, and CM. The Chinese/Taiwanese sample was collected by CS, SJ, CM, ML and HC-K. Translation of Chinese measures was conducted by LQ and ML. Taiwanese translation was conducted by HC-K. All authors worked on the final draft and approved the final version of the manuscript.

## Funding

The position of CM is funded by a Heisenberg grant awarded to him by the German Research Foundation (MO 2363/3-2). JW has a stipend from the German Academic Scholarship Foundation (Studienstiftung des deutschen Volkes).

## Conflict of Interest Statement

The authors declare that the research was conducted in the absence of any commercial or financial relationships that could be construed as a potential conflict of interest.
