# Supplementary material for: Anxiety-Related Coping Styles, Social Support, and Internet Use Disorder
Source: Front Psychiatry. 2019 Sep 24;10:640. doi: 10.3389/fpsyt.2019.00640 (PMC6785757; doi:10.3389/fpsyt.2019.00640)
Supplement: Supplementary file 1 [file DataSheet_1.doc]

**Supplementary Material**

**1. Taiwanese Sample**

In line with the German sample we found acceptable internal consistencies for all measures in Taiwanese language (Cronbach’s alphas: GPIUS2: .927; CAV-E: .743; CAV-P: .751; CAV-T: .828; VIG-E: .681; VIG-P: .735; VIG-T: .785; FERUS social support: .872) in the Taiwanese sample.

**Table I**

Mean scores (standard deviations) of GPIUS2*,* all ABI variables, social support and social network size and percentages for each SNS category for the total Taiwanese sample (N = 104) and for the male and female subsample with mean differences and Cohen’s *d*

|  |  | Total (N= 104) | Male (n = 29) | Female (n= 75) | *MD* | *Cohen’s d* |
| --- | --- | --- | --- | --- | --- | --- |
| GPIUS2  CAV-E  CAV-P  CAV-T  VIG-E  VIG-P  VIG-T  Social support  SNS (score)  SNS (0)  SNS (1)  SNS (2 -5)  SNS (6 – 9)  SNS (10 or more) | | 50.86 (20.07) | 55.41 (17.99) | 49.09 (20.66) | **6.32** | **.32** |
|  |  | 10.69 (3.71) | 10.97 (3.54) | 10.59 (3.79) | 0.38 | .10 |
|  |  | 11.80 (3.87) | 11.59 (3.46) | 11.88 (4.04) | -0.29 | .08 |
|  |  | 22.49 (6.57) | 22.55 (6.17) | 22.47 (6.76) | 0.09 | .01 |
|  |  | 16.70 (2.74) | 16.59 (2.60) | 16.75 (2.81) | -0.16 | .06 |
|  |  | 14.68 (3.41) | 14.21 (3.73) | 14.87 (3.29) | -0.66 | .19 |
|  |  | 31.38 (5.12) | 30.79 (5.68) | 31.61 (4.91) | -0.82 | .16 |
|  |  | 37.61 (6.30) | 35.41 (5.77) | 38.45 (6.33) | **-3.04** | **.49** |
|  |  | 3.64 (0.78) | 3.52 (0.74) | 3.69 (0.79) | **-0.18** | **.23** |
|  |  | 0.00% | 0.00% | 0.00% | - | - |
|  |  | 0.00% | 0.00% | 0.00% | - | - |
|  |  | 53.85% | 62.07% | 50.67% | - | - |
|  |  | 27.88% | 24.14% | 29.33% | - | - |
|  |  | 18.27% | 13.79% | 20.00% | - | - |

Abbreviations: GPIUS2 = Generalized Problematic Internet Use Scale 2; CAV-E = cognitive avoidance (ego-threat); CAV-P = cognitive avoidance (physical threat); CAV-T = cognitive avoidance (total score); VIG-E = vigilance (ego-threat); VIG-P = vigilance (physical threat); VIG-T = vigilance (total score); SNS = social network size, MD = mean difference, bolded values indicate Cohen’s *d* > .20.

In line with the German sample we observed higher GPIUS2 scores and lower social support scores for males than females. But no comparable mean differences were found for vigilance and cognitive avoidance for males and females in the Taiwanese sample.

**Table II**

Spearman correlations between GPIUS2, cognitive avoidance, vigilance, social support, social network size and age in the Taiwanese sample (N = 104)

| Correlation coefficients | | | | | | | | | | |
| --- | --- | --- | --- | --- | --- | --- | --- | --- | --- | --- |
|  | GPIUS2 | CAV-E | CAV-P | CAV-T | VIG-E | VIG-P | VIG-T | Social support | SNS | Age |
| GPIUS2 | 1 | -.145 | .068 | -.039 | .084 | .102 | .121 | -.028 | -.080 | .030 |
| CAV-E |  |  | .496*** | .847*** | -.001 | .246* | .173 | -.039 | -.045 | .076 |
| CAV-P |  |  |  | .860*** | .151 | .006 | .068 | -.123 | -.095 | .132 |
| CAV-T |  |  |  |  | .069 | .133 | .123 | -.101 | -.052 | .093 |
| VIG-E |  |  |  |  |  | .384*** | .782*** | -.045 | -.064 | .002 |
| VIG-P |  |  |  |  |  |  | .860*** | .208* | .109 | -.073 |
| VIG-T |  |  |  |  |  |  |  | .119 | .051 | -.037 |
| Social support |  |  |  |  |  |  |  |  | .453*** | -.087 |
| SNS |  |  |  |  |  |  |  |  |  | -.130 |
| Age |  |  |  |  |  |  |  |  |  | 1 |

Abbreviations: GPIUS2 = Generalized Problematic Internet Use Scale 2; CAV-E = cognitive avoidance (ego-threat); CAV-P = cognitive avoidance (physical threat); CAV-T = cognitive avoidance (total score); VIG-E = vigilance (ego-threat); VIG-P = vigilance (physical threat); VIG-T = vigilance (total score); SNS = social network size

Note: * p < .05; ** p <.01, *** p < .001 (two-tailed tested).

No significant correlations between any of the tested variables and the GPIUS2 scores were found. The direction and size of the correlation patterns of vigilance/cognitive avoidance variables and GPIUS2 scores is mostly comparable to what is observed in the main manuscript for the German sample. Differences in significance likely are attributable to the much lower power in the Taiwanese sample. Perhaps the largest difference to be seen is the non-existent correlation between the variable of social support and GPIUS2 (in the main manuscript r = -.34 and here only r = -.03). In this context, we explicitly refer to the fact that a) future research needs to disentangle the role of actual (offline) social support and virtual (online) social support, because the work by Yeh et al. (2008) demonstrated different associations of both kinds of social support in Taiwanese students. Beyond that and b) some of the FERUS items might not work well in Asian (Taiwanese) populations, because items such as hugging friends or relatives is not a common way to express emotional support in Taiwan. Finally, support from friends and family is mixed in the FERUS self-report, although these forms of support might be differently associated with Internet use disorders.

**2. German Sample**

**2.1 GPIUS2**

**Blom-Transformation**

**Histogram GPIUS2**

**Histogram GPIUS2 Blom-transformed**

**GPIUS 2 subscales**

**Table III**

Spearman correlations between GPIUS2 subscales, cognitive avoidance, vigilance, social support, social network size and age in the German sample (n = 567)

|  | |  | |  | |  | | Correlation coefficients | | | | | | | | | | |
| --- | --- | --- | --- | --- | --- | --- | --- | --- | --- | --- | --- | --- | --- | --- | --- | --- | --- | --- |
|  | POSI | | MO | | CP | | CIU | | NO | CAV-E | CAV-P | CAV-T | VIG-E | VIG-P | VIG-T | Social support | SNS | Age |
| POSI | 1 | | .365*** | | .388*** | | .360*** | | .373*** | -.113** | -.091* | -.122** | .076 | .057 | .075 | -.360*** | -.172*** | -.016 |
| MO |  | |  | | .506*** | | .435*** | | .392*** | -.072 | -.013 | -.060 | .148*** | .137** | .160*** | -.244*** | -.124*** | -.135** |
| CP |  | |  | |  | | .599*** | | .507*** | -.091* | -.059 | -.094* | .145** | .131** | .159*** | -.228*** | -.030 | -.136** |
| CIU |  | |  | |  | |  | | .663*** | -.082 | .053 | -.023 | .115** | .062 | .100* | -.223*** | -.092* | -.154*** |
| NO |  | |  | |  | |  | |  | -.097* | -.006 | -.059 | .102* | .078 | .100* | -.283*** | -.073 | -.069 |
| CAV-E |  | |  | |  | |  | |  |  | .437*** | .866*** | -.373*** | -.172*** | -.318*** | .017 | .024 | .054 |
| CAV-P |  | |  | |  | |  | |  |  |  | .811*** | -.094* | -.388*** | -.277*** | .049 | .073 | -.076 |
| CAV-T |  | |  | |  | |  | |  |  |  |  | -.286*** | -.317*** | -.350*** | .043 | .059 | -.013 |
| VIG-E |  | |  | |  | |  | |  |  |  |  |  | .509*** | .863*** | -.043 | -.068 | -.017 |
| VIG-P |  | |  | |  | |  | |  |  |  |  |  |  | .865*** | -.039 | -.088* | -.018 |
| VIG-T |  | |  | |  | |  | |  |  |  |  |  |  |  | -.044 | -.088* | -.016 |
| Social support |  | |  | |  | |  | |  |  |  |  |  |  |  |  | .345*** | -.039 |
| SNS |  | |  | |  | |  | |  |  |  |  |  |  |  |  |  | -.057 |
| Age |  | |  | |  | |  | |  |  |  |  |  |  |  |  |  | 1 |

Abbreviations: POSI = Preference for Online Social Interaction; MO = Mood regulation; CP = Cognitive Preoccupation; CIU = Compulsive Internet Use; NO = Negative Outcome; CAV-E = cognitive avoidance (ego-threat); CAV-P = cognitive avoidance (physical threat); CAV-T = cognitive avoidance (total score); VIG-E = vigilance (ego-threat); VIG-P = vigilance (physical threat); VIG-T = vigilance (total score); SNS = social network size

Note: * p < .05; ** p <.01, *** p < .001 (two-tailed tested).

**Hierarchical linear Regression – Model 1**

**Table IV Model 1 [Block 1]**

| Variable |  | *B* | *SE of B* | *Beta* | *P* | *CI* |
| --- | --- | --- | --- | --- | --- | --- |
| **Age^a^** |  | -.323 | .039 | -.328 | <.001 | [-.045; -032] |
| **Gender^a^** |  | -.272 | .086 | -.125 | .002 | [-.435; -.118] |

Note: All predictors except gender in z-standardized form, gender coded: 1 = male, 2 = female, significant predictors presented in bold letters, ^a^ significant after bootstrapping analysis.

**Table IV Model 1 [Block 1 + Block 2]**

| Variable |  | *B* | *SE of B* | *Beta* | *P* | *CI* |
| --- | --- | --- | --- | --- | --- | --- |
| **Age^a^** |  | -.323 | .036 | -.327 | <.001 | [-.044; -032] |
| **Gender^a^** |  | -.253 | .084 | -.116 | .003 | [-.411; -.099] |
| **CAV-E** |  | -.077 | .039 | -.078 | .049 | [-.000; .182] |
| **VIG-E** |  | .091 | .045 | .092 | .044 | [.000; .190] |
| **VIG-P^a^** |  | .094 | .043 | .095 | .031 | [-.162; .008] |
| **Social Support^a^** |  | -.320 | .037 | -.325 | <.001 | [-.408; -.242] |

Abbreviations: CAV-E = cognitive avoidance (ego-threat); VIG-E = vigilance (ego-threat); VIG-P = vigilance (physical threat)

Note: All predictors except gender in z-standardized form, gender coded: 1 = male, 2 = female, significant predictors presented in bold letters, ^a^ significant after bootstrapping analysis.

**Hierarchical linear Regression – Model 2**

**Table V Model 2 [Block 1]**

| Variable |  | *B* | *SE of B* | *Beta* | *P* | *CI* |
| --- | --- | --- | --- | --- | --- | --- |
| **Age^a^** |  | -.323 | .039 | -.328 | <.001 | [-.045; -.032] |
| **Gender^a^** |  | -.272 | .086 | -.125 | .002 | [-.441; -.120] |

Note: All predictors except gender in z-standardized form, gender coded: 1 = male, 2 = female, significant predictors presented in bold letters, ^a^ significant after bootstrapping analysis.

**Table VI Model 2 [Block 1 + Block 2 + Block 3]**

| Variable |  | *B* | *SE of B* | *Beta* | *P* | *CI* |
| --- | --- | --- | --- | --- | --- | --- |
| **Age^a^** |  | -.340 | .038 | -.345 | <.001 | [-.048; -.031] |
| **Gender^a^** |  | -.400 | .089 | -.184 | <.001 | [-.562; -.265] |
| CAV-E |  | .119 | .815 | .120 | .884 | [-2.401; .835] |
| VIG-E |  | -.214 | 1.503 | -.217 | .887 | [-1.660; 1.589] |
| VIG-P |  | .048 | 1.036 | .049 | .963 | [-.977; 1.284] |
| SNS (1) |  | -.584 | 1.068 | -.126 | .585 | [-3.247; .575] |
| SNS (2-5) |  | -.777 | 1.052 | -.382 | .461 | [-3.512; .519] |
| SNS (6 -9) |  | -.972 | 1.054 | -.424 | .357 | [-3.711; .326] |
| SNS (10 or more) |  | -.891 | 1.062 | -.234 | .401 | [-3.526; .252] |
| SNS (1) x CAV_E |  | -.304 | .842 | -.061 | .718 | [-1.284; 2.544] |
| SNS (2-5) x CAV_E |  | -.211 | .817 | -.178 | .796 | [-.977; 2.437] |
| SNS (6 - 9) x CAV_E |  | -.069 | .820 | -.030 | .933 | [-.937; 2.727] |
| SNS (10 or more) x CAV_E |  | -.278 | .829 | -.074 | .738 | [-1.336; 2.573] |
| SNS (1) x VIG_E |  | .328 | 1.514 | .074 | .829 | [-1.665; 2.075] |
| SNS (2-5) x VIG_E |  | .278 | 1.504 | .224 | .853 | [-1.581; 1.767] |
| SNS (6 -9) x VIG_E |  | .428 | 1.505 | .220 | .776 | [-1.510; 2.004] |
| SNS (10 or more) x VIG_E |  | .282 | 1.513 | .069 | .852 | [-1.796; 1.976] |
| SNS (1) x VIG_P |  | .471 | 1.065 | .085 | .659 | [-.844; 1.511] |
| SNS (2-5) x VIG_P |  | .035 | 1.038 | .030 | .973 | [-1.197; 1.087] |
| SNS (6 -9) x VIG_P |  | .059 | 1.041 | .027 | .955 | [-1.149; 1.101] |
| SNS (10 or more) x VIG_P |  | .152 | 1.048 | .041 | .885 | [-1.144; 1.224] |

Abbreviations: CAV-E = cognitive avoidance (ego-threat); VIG-E = vigilance (ego-threat); VIG-P = vigilance (physical threat); SNS = social network size

Note: All predictors except gender in z-standardized form, gender coded: 1 = male, 2 = female, significant predictors presented in bold letters, ^a^ significant after bootstrapping analysis.

**Reference**

Yeh, Y. C., Ko, H. C., Wu, J. Y. W., & Cheng, C. P. (2008). Gender differences in relationships of actual and virtual social support to Internet addiction mediated through depressive symptoms among college students in Taiwan. *CyberPsychology & Behavior*, *11*(4), 485-487.
